# Beyond the effects of HIV infection and integrase inhibitors-based therapies on oral bacteriome

**DOI:** 10.1038/s41598-023-41434-5

**Published:** 2023-08-31

**Authors:** Pablo Villoslada-Blanco, Patricia Pérez-Matute, Emma Recio-Fernández, María Íñiguez, Pilar Blanco-Navarrete, Luis Metola, Valvanera Ibarra, Jorge Alba, María de Toro, José A. Oteo

**Affiliations:** 1 Infectious Diseases, Microbiota and Metabolism Unit, Infectious Diseases Department, Center for Biomedical Research of La Rioja (CIBIR), C/Piqueras 98, CIBIR Building, Third Floor, 26006 Logroño, La Rioja Spain; 2 Centro de Salud Siete Infantes de Lara, Logroño, La Rioja Spain; 3 Infectious Diseases Department, Hospital Universitario San Pedro, Logroño, La Rioja Spain; 4 Genomics and Bioinformatics Platform, Center for Biomedical Research of La Rioja (CIBIR), Logroño, La Rioja Spain

**Keywords:** Microbiology, Diseases

## Abstract

Oral microbiome is the second largest microbial community in humans after gut. Human immunodeficiency virus (HIV) infection triggers an impairment of the immune system which could favour the growth and the colonization of pathogens in the oral cavity, and this dysbiosis has been associated with oral manifestations that worsen the quality of life of these patients. Antiretroviral therapy (ART) could also drive changes in specific oral bacterial taxa associated with such periodontal diseases. Integrase strand transfer inhibitors (INSTIs), therapy of choice in the treatment of naive HIV-patients, are able to reverse the impact of HIV infection on systemic inflammation, gut permeability, and gut bacterial diversity/richness. The objective of this study was to analyse the effects of HIV infection per se and INSTIs on salivary bacteriome composition, taking into consideration other factors such as smoking, that could also have a significant impact on oral microbiome. To accomplish this objective, 26 non-HIV-infected volunteers and 30 HIV-infected patients (15 naive and 15 under INSTIs-regimen) were recruited. Salivary samples were collected to measure lysozyme levels. Oral bacteriome composition was analysed using 16S rRNA gene sequencing. Naive HIV-infected patients showed statistically higher levels of lysozyme compared to controls (*p* < 0.001) and INSTIs-treated patients (*p* < 0.05). Our study was unable to detect differences in α nor β-diversity among the three groups analysed, although significant differences in the abundance of some bacterial taxonomical orders were detected (higher abundance in the phylum Pseudomonadota, in the order Acholeplasmatales, and in the genera *Ezakiella* and *Acholeplasma* in the naive group compared to controls; and higher abundance in the phylum Mycoplasmatota, in the order Acholeplasmatales, and in the genera *Acholeplasma* and *uncultured Eubacteriaceae bacterium* in the INTIs-treated HIV-infected patients compared to controls). These differences seem to be partially independent of smoking habit. HIV infection and INSTIs effects on oral microbiota seem not to be very potent, probably due to the modulation of other factors such as smoking and the greatest outward exposure of the oral cavity.

## Introduction

HIV infection triggers an impairment of the immune system which could favour the growth and the colonization of pathogens in the oral cavity. In fact, this dysbiosis has been associated with oral manifestations such as oropharyngeal candidiasis^1–3^. Besides, antiretroviral treatment (ART) itself can increase oral microbial translocation due to the inhibition of epithelial cell repair and proliferation^4, 5^. HIV and ART drive changes in specific bacterial taxa in oral microbiome associated with periodontal disease^6^. In fact, it has been recently reported that shifts in oral microbiome after ART initiation are complex, and may play an important role in immune function and inflammatory disease^6^. The analysis of oral microbiota through supra and subgingival plaque samples is often time-consuming^7, 8^. Because of that, saliva-based analysis has gained considerable attention since it is simple, non-invasive, and inexpensive^9–11^.

Previous works from our group demonstrated that ARTs based on integrase strand transfer inhibitors (INSTIs) were associated with levels of systemic inflammation, bacterial translocation, and microbial diversity similar to those observed in uninfected controls^12^. Besides, these studies showed a clear impact of HIV-infection and INSTIs-based treatments on gut bacteriome^13^ and virome^14^. However, how long-term ART, and specifically INSTIs, modulate oral microbiota in HIV-infected patients and the implications of these effects on health deserve further research. Thus, the objective of this work was to analyse the effects of HIV infection and INSTIs-based therapies in first line of treatment on salivary bacteriome composition. Besides, seeing that some habits such as smoking could alter oral microbiota^15^, we also investigated the impact of smoking in oral microbiota composition of HIV-infected patients with and without INSTIs-based treatments.

## Methods

### Patient recruitment

HIV-infected patients (naive and under ART) as well as the non-infected volunteers used as “healthy” controls were recruited from the Infectious Diseases Department at Hospital Universitario San Pedro (HUSP) and from the Health Care Center “Siete Infantes de Lara” (Logroño, Spain) from March 2019 to February 2021, as previously described^13, 14^. Supplementary Fig. 1 shows a flowchart of patient recruitment, and the characteristics of these groups can be consulted in references^13, 14^.

### Saliva samples collection and lysozyme levels measurement

Fresh saliva samples were received at CIBIR, aliquoted in tubes (approximately 500 μl) and stored at − 80 °C for further analysis. After defrosting, lysozyme concentration in saliva was measured through enzyme-linked immunosorbent assay (ELISA) from ABCAM (Ab108880) according to the manufacturer’s instructions. The intra-assay and the inter-assay coefficient of variation (CV) expressed as percentage are 4.7% and 9.5%, respectively.

### DNA extraction from saliva and 16S rRNA gene sequencing

After defrosting, DNA was extracted using a modified version of the DNeasy^®^ Blood and Tissue kit (Qiagen, Venlo, The Netherlands). In this protocol, 250 µl of saliva is transferred to an Eppendorf tube. Then, 1 ml of PBS is added and the mixture is centrifuged at 1800 g for 5 min. The supernatant is decanted and the pellet is resuspended in 180 µl of PBS and transferred to another Eppendorf tube. Next, 25 µl of Proteinase K and 200 µl of Buffer AL are added, followed by vortexing for 20 s and centrifugation. The mixture is incubated at 56 °C for 10 min. After that, 200 µl of ethanol is added, vortexed for 20 s, and centrifuged. The resulting solution is transferred to a column and centrifuged, at 6000 g for 1 min and the collected tube with the filtrate is discarded. The column is then placed in a new collection tube, 500 µl of Buffer AW1 is added and centrifuged, and the collected tube with the filtrate is discarded. Then, the column is again placed in a new collection tube, 500 µl of Buffer AW2 is added and centrifuged at 20,000 g for 3 min, and the collected tube with the filtrate is discarded. Finally, the column is placed in a new Eppendorf tube, 50 µl of Buffer AE is added, and incubated at room temperature for 1 min. The mixture is then centrifuged at 6000 g for 1 min and the elution process is repeated. After this process, purity, concentration, and quality were determined by a Qubit 3.0 fluorometer (kit dsDNA HS, Thermo Fisher Scientific, MA, USA) and a Fragment Analyzer (HS Genomic DNA 50 Kb kit, Agilent, USA). Then, samples were amplified for the 16S rRNA hypervariable regions V3-V4^16^ and sequencing was performed using an Illumina sequencer (MiSeq, 2 × 300 pb, paired end) at the Genomics & Bioinformatics Core Facility at CIBIR.

The first step in computational analysis was to check the quality of reads by the quality control tool FastQC program^17^. Then, the Qiime2 pipeline^18^ was used along the bioinformatic analysis. Firstly, the raw sequences already demultiplexed (mapping the barcodes to the samples they belong) by the Illumina sequencer were denoised using the DADA2 software^19^. Specifically, it was performed the trimming of sequencing adapters and primer regions, the filtering of noisy reads, the dereplicate of our sequences to reduce repetition, the joint of paired reads, the identification of amplicon sequence variants (ASVs), and the elimination of chimeras. Secondly, we used the SILVA database^20^ trained with the V3-V4 amplification primers using during the wet-lab process to do the taxonomic assignation. The alpha and beta diversity were analysed: α-diversity is a measure of sample-level species richness, whereas β-diversity describes inter-subject similarity of microbial composition and facilitates the identification of broad differences between samples. The measure of α-diversity was analysed using *Observed Features*, *Chao1 index*, *Fisher’s alpha*, *Pielou’s index*, *Shannon index*, and *Simpson index* and differences between groups were assessed through Kruskal–Wallis test. *Observed features*, *Chao1 index* and *Fisher’s alpha* are based in richness, *Pielou’s index* is based in evenness and *Shannon index* and *Simpson index* are based in diversity (richness + evenness). The measure of β-diversity was analysed using PERMANOVA (999 permutations) on Bray Curtis metric and visualized using Principal Coordinate Analysis (PCoA) by R software (version 4.0.5) and R Studio (version 1.4.1105). Finally, the analysis of the differential composition of bacteria was carried out with the ANCOM methodology at phylum, order, and genus taxonomic levels. This methodology accounts for the underlying structure in the data and is widely used for comparing the composition of microbiomes in two or more populations, with no assumptions of population distribution^21^. ANCOM just runs a bunch of pairwise tests for the following sub-hypothesis:

$${H}_{0(ij)}:mean\left(log\frac{{x}_{i}}{{x}_{j}}\right)=mean\left(log\frac{{y}_{i}}{{y}_{j}}\right)$$

where ${x}_{i}$ denotates the ith ASV abundance from sample x, ${x}_{j}$ denotates de jth ASV abundance from sample x, ${y}_{i}$ denotates the ith ASV abundance from sample y and ${y}_{j}$ denotates de jth ASV abundance from sample y. The W value is just a count of the number of times that ${H}_{0(ij)}$ is rejected for the ith ASV.

### Statistical analysis

Characteristics of the population and lysozyme levels are presented as mean ± standard error of the man (SEM). Categorical variables were analysed using the Chi-square or Fisher’s exact test. Normal distribution of quantitative variables was checked using the Shapiro–Wilk test. Comparison between two groups were performed using unpaired *t* test or Mann–Whitney U test depending on the normality of the data. Comparison between three or more groups were analysed using ANOVA followed by Tukey post-hoc regardless the normality of the data. *p* values < 0.05 and false discovery rates (FDRs) < 0.05 were considered as statistically significant. Statistical analysis was performed using R software (version 4.0.5) and R Studio (version 1.4.1105).

### Compliance with ethics guidelines

This study was performed following the Helsinki Declaration and was approved by the Committee for Ethics in Drug Research in La Rioja (CEImLAR) (28 February 2019, reference number 349). All participants provided their written informed consent.

## Results

### Clinical and demographical characteristics of participants

The main characteristics of the recruited population (Supplementary Table 1 and Supplementary Table 2) were previously described^13, 14^. Of note, smoking habits were higher in the naive group and INSTIs-treated group compared to controls (11.54 vs. 46.67% and 66.67%, respectively). On the other hand, none of the individuals recruited reported periodontal diseases. Finally, none of the patients reported differential dietary patterns.

### Salivary lysozyme levels

**Figure 1:**
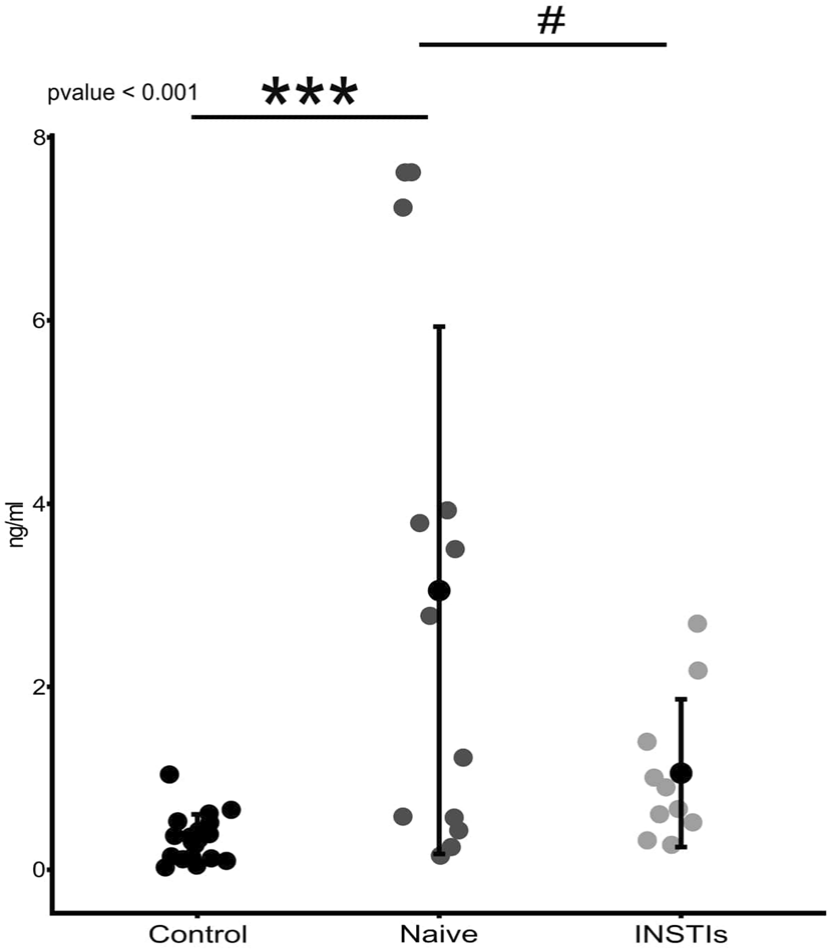
Levels of lysozyme in the studied population comparing control group, naive group, and INSTIs-treated group. ***p < 0.001 versus control and ^#^*p* < 0.05 versus INSTIs. *INSTIs* integrase strand transfer inhibitors-based treatment.

A statistically significant increase in salivary lysozyme concentrations was observed in naive HIV-infected patients compared to the control population (*p* < 0.001) and also compared to INSTIs-treated patients (*p* < 0.05) (Fig. 1). No statistically significant difference was detected between the control and the INSTIs-treated group (Fig. 1).

### DNA extractions, libraries preparation and sequencing

After DNA extraction, all samples had a DNA concentration between 11.40 and 336.00 ng/μl with a mean of 86.77 ng/μl and a median of 73.70 ng/μl. A total of 12.5 ng of each one was used for the amplification PCR, the first step at the library preparation protocol. Besides, attending to the DNA quality, measured as DNA integrity through the GQN parameter in the FA system, all samples were ranked between 0.6 and 3.6 values. In samples where a specific fragment of 450–500 bp was amplified, the assessment of genomic integrity may not be as significant since the focus is on the amplification of a specific target region rather than the overall integrity of the genome. As an intermediate step, after the amplicon PCR, but before the indexing step, the presence of the amplicon was verified using Fragment Analyzer (Amplicon Library kit, 35–5000 bp, AATI), resulting in fragments ranging from 500 to 540 bp in size. This step confirmed the successful amplification of the target region and ensured the presence of the desired amplicon for further analysis or sequencing. After library preparation, all the final libraries were quantified once again using Qubit fluorometry and qualified using Fragment Analyzer (Amplicon Library kit, 35–5000 bp, AATI) to determine the average amplicon size. This information (concentration and medium library size) allowed us to subsequently normalize the libraries to the same concentration (nM) in order to prepare an equimolar pool at 10 pM. In this case, the libraries had a concentration ranging from 31 to 210 ng/uL, with a mean value of 94.80 ng/uL, as determined by fluorometry. Additionally, the average amplicon size ranged from 600 to 630 bp, taking into account that this size includes 120 bp corresponding to the sequencing adapters included in the library preparation for the V3-V4 amplicon by PCR. The individual average amplicon size with the library concentration (ng/uL) was used to calculate the molarity of each sample, normalized at 10 nM and a final equimolar pool at 10 pM, that was introduced into the MiSeq sequencer.

The raw sequencing reads obtained from the saliva samples in this study ranged from a minimum of 134,247 reads to a maximum of 396,314 reads. After adapter trimming, removal of low-quality regions, and processing using the DADA2 protocol, a total of between 66,634 and 187,188 reads (corresponding to a percentage range relative to the initial 38.33–50.34% of the initial reads) were maintained.

### Salivary bacteriome diversity and composition

#### Alpha diversity

No statistical differences were observed on α-diversity of oral bacteriome (*Observed features*, *Chao1 index*, *Fisher’s alpha*, *Pielou’s evenness, Simpson index*, and *Shannon index*) (Supplementary Fig. 2).

#### Beta diversity

The analysis of β-diversity did not either reveal a different clustering pattern between the three groups analysed (Supplementary Fig. 3).

#### Differential abundance

Concerning oral microbiota composition and/or abundance, a total of 20 phyla and 68 orders were detected. The three most abundant phyla in saliva were Bacteroidota (12.61–64.16%), Bacillota (7.37–66.14%), and Pseudomonadota (0.12–54.40%). In the order level, Bacteroidales (10.05–63.13%), Lactobacillales (2.65–56.11%), and Betaproteobacteriales (0.04–42.54%) were the most abundant.

When comparing controls to naive patients, a significant higher abundance in the phylum Pseudomonadota, in the order Acholeplasmatales (phylum Bacillota), and in the genera *Ezakiella* (order Tissierellia, phylum Bacillota) and *Acholeplasma* (order Acholeplasmatales, phylum Bacillota) were observed in the naive group compared to controls (Table 1). On the other hand, when controls were compared against INSTIs-treated HIV-infected patients, a higher abundance in the phylum Mycoplasmatota, in the order Acholeplasmatales (phylum Bacillota), and in the genera *Acholeplasma* (order Acholeplasmatales, phylum Bacillota) and *uncultured Eubacteriaceae bacterium* (order Eubacteriales, phylum Bacillota) were detected in the INSTIs-treated HIV-infected patients (Table 1). No differences were observed between both HIV-infected groups. No lower abundances were observed in HIV-infected patients.

**Table 1:** Bacterial taxonomical orders differentially abundant in the saliva of the studied population.

	Control versus					
	Naive			INSTIs		
	Category	Taxonomic group	W	Category	Taxonomic group	W
↑	Phylum	Pseudomonadota	13	Phylum	Mycoplasmatota	19
	Order	Acholeplasmatales	66	Order	Acholeplasmatales	57
	Genus	*Acholeplasma*	124	Genus	*Acholeplasma*	178
		*Ezakiella*	130		*uncultured Eubacteriaceae bacterium*	162

*INSTIs* integrase strand transfer inhibitors-based treatment.

### Effects of smoking on oral microbiota

As smoking has an impact on oral microbiota^22^, we assessed if smoking habits could modulate those actions exerted by HIV infection per se and/or INSTIs. We revealed that oral microbiota coming from smoking individuals (irrespectively of their HIV status) showed a statistically significant increase in all α-diversity indexes analysed (*p* < 0.01 for *Observed features*, *Chao1 index,* and *Fisher’s alpha* and *p* < 0.05 *Pielou’s evenness*, *Shannon index,* and *Simpson index*) compared to non-smokers (Supplementary Fig. 4). Besides, the analysis of β-diversity revealed a different clustering between the two groups (*p* < 0.001) (Supplementary Fig. 5) suggesting a clear effect of tobacco on oral microbiota composition/community.

**Figure 2:**
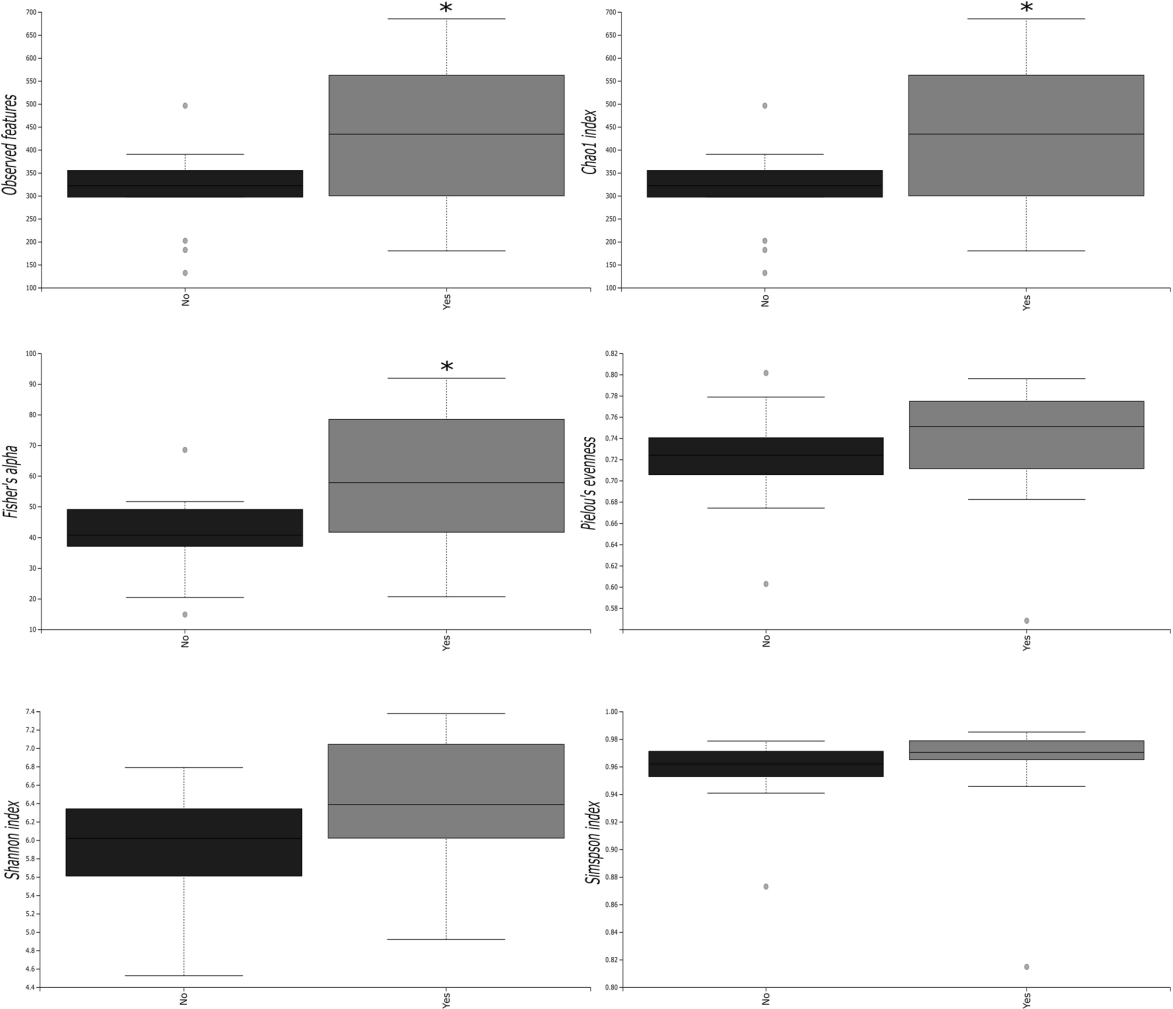
Different indexes of α-diversity from bacteria in salivary samples of HIV-infected patients regarding smoking habit. *p < 0.05 versus non-smokers.

**Figure 3:**
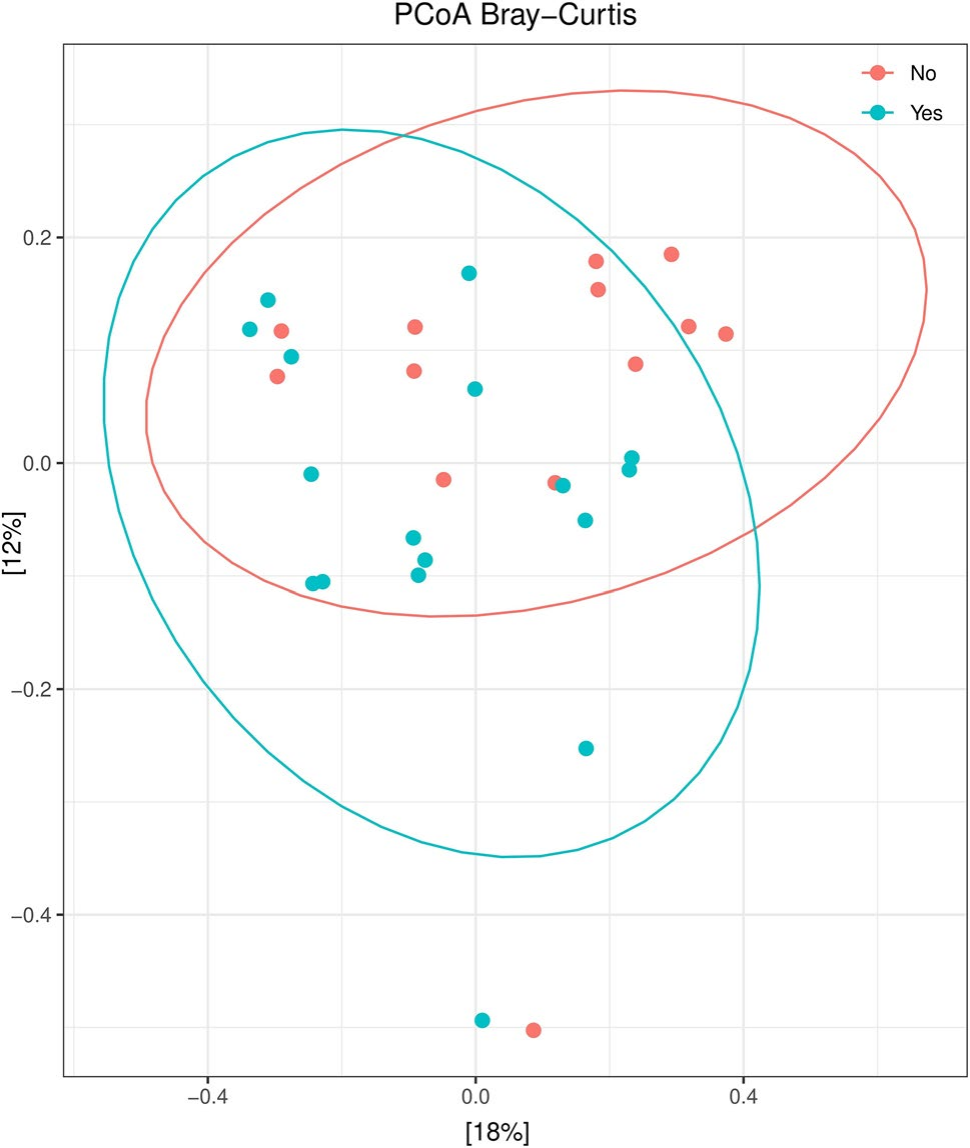
PCoAs from bacteria in salivary samples from HIV-infected patients regarding smoking habit (accounting for 30% of the total variation [Component 1 = 18% and Component 2 = 12%]). Results are plotted according to the first two principal components. Each circle represents a sample: red circles represent the non-smoking individuals and blue circles represent the smoking individuals. The clustering of samples is represented by their respective 95% confidence interval ellipse. *p < 0.05 smokers versus non-smokers.

Similarly, when only HIV-infected patients were split into two groups according to their smoking habits (and regardless ART), those samples from smoking individuals presented a statistically significant increase in the richness indexes analysed (*p* < 0.05 for *Observed features*, *Chao1 index,* and *Fisher’s alpha*) (Fig. 2) compared to non-smoking HIV-infected population. Moreover, the analysis of β-diversity revealed a different clustering between the two groups (*p* < 0.05) (Fig. 3).

Considering the clear impact of smoking on oral microbiota, we next analysed the controls, naive HIV-infected patients, and INSTIs-treated patients taking into account their smoking status, comparing firstly the non-smokers and, secondly, the smokers.

No differences were detected in the α-diversity of the non-smoking population (Supplementary Fig. 6). Besides, the analysis of β-diversity did not either reveal a different clustering between the three groups (Supplementary Fig. 7). However, the analysis of differential abundance revealed some taxonomical orders differentially represented between the groups, although the magnitudes of such differences were not very potent. Specifically, the INSTIs-treated group showed a higher abundance of phylum Mycoplasmatota (W = 1) and a lower abundance of phyla Epsilonbacteraeota (W = 4), Bacteroidota (W = 1), Spirochaetota (W = 1), Cemmatimonadetes (W = 1), Chlamydiota (W = 1), Acidobacteriota (W = 1), and Campylobacterota (W = 1) and of order Campylobacterales (W = 10) when compared to controls. On the other hand, a higher abundance of phyla Epsilonbacteraeota (W = 2) and Bacteroidota (W = 1) were observed in the INSTIs-treated group compared to naive group.

On the other hand, no significant differences were observed in either α-diversity or β-diversity or differential abundance when only smokers were analysed (Supplementary Fig. 8 and 9).

## Discussion

Contrary to the results observed in our previous works focused on gut microbiota^13, 14^, the effects of HIV infection and INSTIs on oral microbiota seem to be not very significant, probably due to the modulation of other factors such as smoking, and the greatest outward exposure of the oral cavity.

Lysozyme is an antimicrobial protein, expressed by various cells including neutrophils, macrophages, and epithelial cells. It is abundant in saliva and plays an important role in the host constitutive defense system^23^. Previous studies have revealed an increase in salivary lysozyme levels in immunocompromised patients^24, 25^, which could serve as a compensatory mechanism to alleviate the decrease in the number of CD4 T lymphocytes. Besides, it was revealed that lysozyme from chicken egg white, human milk, and human neutrophils possesses antiviral activity against HIV-infection in vitro^26^. Considering that our study was focused on oral microbiota, we believed interesting to quantify this marker as an indirect measure of the HIV-infection and more specifically, the mechanisms that are set in motion to try to counteract the infection action and the potential association with oral microbiota. In fact, we observed a statistically significant increase of salivary lysozyme concentration in naive HIV-infected patients compared to controls and INSTIs in parallel with the decrease observed in CD4 T lymphocytes in these patients, suggesting a possible relationship between lysozyme, infection, and immunological response. Interestingly, INSTI-treated patients showed a significant decrease in the salivary levels of this enzyme along with the concomitant increase of CD4 T lymphocytes. To our knowledge, this is the first study that explored the effects of INSTIs on salivary lysozyme concentrations. Our results could highlight the potential usage of this enzyme as a non-invasive biomarker of the immunological recovery observed after INSTIs, but more studies are needed in this regard.

Our study was unable to detect differences in α nor in β-diversity associated with HIV infection and/or INSTIs-based treatment. Similarly, Presti et al.^6^, were unable to detect differences in α nor in β-diversity between the naive group and the ART-treated group (treated with EFV/FTC/tenofovir(TDF)). Imahashi et al.^27^, did not either show differences in species richness between HIV negative people and ART-treated HIV-infected subjects. However, Li et al.^28^, revealed a statistically significant decrease in *Chao1 index* and *Shannon index* between the naive group and the control group and also a different clustering between both groups. The differences could be explained by the fact that the populations among studies were different. In fact, in the study of Li et al.^28^, only HIV-infected MSM patients from Beijing were included, whereas in our study the percentage of MSM were 60.00% in the naive group and only 46.67% in the ART-treated group. In addition, only 15 HIV-infected patients in our study were not Caucasians (none of them from Asia). Thus, the potential influence of the origin of subjects (in terms of “dietary habits”) and/or the sexual orientation and mode of transmission of HIV infection in the composition of oral microbiota needs to be further investigated. In addition, the hypervariable regions of the 16S rRNA gene analysed were also different between both studies (V4-V5 vs. V3-V4 in our study).

In our study, the three most abundant phyla were Bacteroidetes (now known as Bacteroidota), Firmicutes (now known as Bacillota), and Pseudomonadota, which correlates with the results from Li et al.^28^ and Presti et al.^6^. We observed some taxa increased in the naive group and in the INSTIs-treated group compared to controls. It is worth mentioning the increase observed in the genus *Acholeplasma* in HIV-infected patients irrespectively of ART. It has been demonstrated that *Acholeplasma laidlawii* could facilitate the entry of HIV infection into cells through the bind to its glycoglycerolipids^29^ and treatment is not able to reduce its presence. The clinical impact (systemic and locally) of these higher abundances should be deeply evaluated.

We also observed that smoking influences salivary bacteriome diversity and composition, as previously revealed by Gopinath et al.^15^, in the general population. However, and despite this fact, we also observed that the impact of HIV infection and INSTIs on oral microbiota were partially independent of smoking since no effects were observed in either α-diversity or β-diversity in both smokers and non-smokers separately analysed. Of note, some differences (not very statistically potent according to the W value) were detected in the abundance of some bacterial taxonomical orders in the non-smoking population whereas no modifications were observed in the smokers suggesting that smoking could modulate or even “buffer/mask” the effects of HIV and INSTIs on oral microbiota. In addition, the less potent effects observed on oral microbiota could be explained by the fact that the oral cavity presents a higher interindividual variability as it is more exposed to environment/diet. Besides, ART pills do not dissolve in saliva, so they have more a systemic effect than a local effect in oral cavity.

This study has some limitations. Some of the patients were recruited during COVID-19 pandemic. However, none of them reported symptoms related to COVID-19 before sample collection and they were not vaccinated in the previous month. Besides, the number of patients in each group is small (what increases the probability of type-II error), but large enough to detect differences among groups. Moreover, some differences between the controls and HIV-infected groups were detected, such as gender, age, and smoking habits, all of them factors that could have an impact on microbiota composition. However, the two HIV-infected groups were well-balanced in terms of these factors. Besides, saliva sampling, although being practical and non-invasive, may not capture the complete diversity of anaerobic bacteria in the mouth, so it cannot be ruled out that other methods will give different results. Finally, patients fulfilled a survey focused on dietary habits/patterns to detect those habits that could have an impact on microbiota, such as veganism or excessive consumptions of prebiotics and/or probiotics. None of the patients reported such differential habits.

## Conclusions

Lysozyme concentration is elevated in naive patients compared to control population suggesting a compensatory mechanism to alleviate the decrease in the number of CD4 T lymphocytes. In fact, INSTI-treatment increased CD4 T cells in the patients and, at the same time, diminished the levels of lysozyme corroborating the association among lysozyme-inmune response and infection. On the other hand, HIV infection and INSTIs impact on oral microbiota was not very potent, probably due to the modulation of other factors such as smoking and the greatest outward exposure of the oral cavity. In fact, smoking increases salivary bacteriome diversity and it also influences bacteriome composition. Therefore, smoking could modulate or even “buffer/mask” the effects of HIV and INSTIs on oral microbiota and this could be the reason for the mild effects observed on salivary microbiome.

### Supplementary Information

Supplementary Information.

## Data Availability

The datasets generated during and/or analysed during the current study are available in the NCBI SRA repository, http://www.ncbi.nlm.nih.gov/bioproject/819232, and also available from corresponding author on reasonable request.
