# Supplementary material for: Beyond the effects of HIV infection and integrase inhibitors-based therapies on oral bacteriome
Source: Sci Rep. 2023 Aug 31;13:14327. doi: 10.1038/s41598-023-41434-5 (PMC10471600; doi:10.1038/s41598-023-41434-5)
Supplement: Supplementary file 1 — Supplementary Information. [file 41598_2023_41434_MOESM1_ESM.docx]

**Supplementary Material for**

**Beyond the effects of HIV infection and integrase strand transfer inhibitors-based therapies on the oral bacteriome**

Villoslada-Blanco Pablo^1^, Pérez-Matute Patricia^1^*, Recio-Fernández Emma^1^, Íñiguez María^1^, Blanco-Navarrete Pilar^2^, Metola Luis^3^, Ibarra Valvanera^3^, Alba Jorge^3^, de Toro María^4^, Oteo José A^1,3^.

^1^Infectous Diseases, Microbiota and Metabolism Unit. Infectious Diseases Department. Center for Biomedical Research of La Rioja (CIBIR), Logroño, La Rioja, Spain.

^2^Centro de Salud Siete Infantes de Lara, Logroño, La Rioja, Spain.

^3^Infectious Diseases Department. Hospital Universitario San Pedro, Logroño, La Rioja, Spain.

^4^Genomics and Bioinformatics Platform. Center for Biomedical Research of La Rioja (CIBIR), Logroño, La Rioja, Spain.

^§^Corresponding author: Patricia Pérez-Matute

Infectious Diseases, Microbiota and Metabolism Unit. Infectious Diseases Department, Center for Biomedical Research of La Rioja (CIBIR), Logroño, La Rioja, Spain.

C/Piqueras 98

CIBIR building, third floor

26006 Logroño (La Rioja)

[cpperez@riojasalud.es](mailto:cpperez@riojasalud.es)

**Supplementary Table 1**

**Supplementary Table 2**

**Supplementary Figure 1**

**Supplementary Figure 2**

**Supplementary Figure 3**

**Supplementary Figure 4**

**Supplementary Figure 5**

**Supplementary Figure 6**

**Supplementary Figure 7**

**Supplementary Figure 8**

**Supplementary Figure 9**

**Supplementary Table 1.** Characteristics of healthy uninfected controls and HIV-infected patients (naive and under INSTIs).

|  | **Control** | **Naive** | **INSTIs-treated** | **p value** |
| --- | --- | --- | --- | --- |
| **Number of patients** | 26 | 15 | 15 | - |
| **Gender (men)** | 9/26 (34.62%) | 13/15 (86.67%) ** | 12/15 (80.00%) ** | **0.002** |
| **Age (years)** | 43.58±2.31 | 33.87±2.85 * | 43.67±3.39 | **0.033** |
| **BMI (kg/m**²) | 24.30±0.69 | 23.23±1.05 | 23.51±0.85 | 0.616 |
| **Waist circumference (cm)** | 85.35±2.55 | 83.83±2.86 | 85.13±2.15 | 0.916 |
| **Systolic blood pressure (mmHg)** | 120.58±2.76 | 135.67±5.67 * | 129.73±6.02 | **0.050** |
| **Diastolic blood pressure (mmHg)** | 72.19±1.94 | 81.87±3.31 * | 77.80±3.24 | **0.035** |
| **Alcohol active** | 3/26 (11.54%) | 0/15 (0.00%) | 1/15 (6.67%) | 0.578 |
| **Smoking active** | 3/26 (11.54%) | 7/15 (46.67%) * | 10/15 (66.67%) *** | **0.001** |

Qualitative variables are represented in percentage while quantitative variables are represented as mean ± standard error mean. P value refers to the comparation between two (naive *vs.* INSTIs) or three (control *vs.* naive *vs.* INSTIs) groups, as appropriate. Statistically significant p values are in bold. Asterisks indicate statistically significant differences with respect to control group (*p<0.05, **p<0.01 and ***p<0.001). BMI (body mass index), INSTIs (integrase strand transfer inhibitors-based treatment).

**Supplementary Table 2.** Characteristics of HIV-infected patients (naive and under INSTIs).

|  | **Naive** | **INSTIs-treated** | **p value** |
| --- | --- | --- | --- |
| **Basal CD4 (cells/µl)** | 464.07±76.46 | 850.53±101.68 | **0.006** |
| **Nadir CD4 (cells/ µl)** | 464.07±76.46 | 526.53 ± 56.30 | 0.517 |
| **CD4/CD8 ratio** | 0.53±0.13 | 0.84±0.10 | **0.027** |
| **Mode of transmission** | HS: 6/15 (40.00%) | HS: 7/15 (46.67%) | 0.716 |
|  | MSM: 9/15 (60.00%) | MSM: 7/15 (46.67%) |  |
|  | Parenteral: 0/15 (0.00%) | Parenteral: 1/15 (6.66%) |  |
| **AIDS** | 1/15 (6.67%) | 0/15 (0.00%) | 1 |
| **Coinfection with hepatitis C virus** | 0/15 (0.00%) | 2/15 (13.33%) | 0.483 |
| **Coinfection with hepatitis B virus** | 0/15 (0.00%) | 0/15 (0.00%) | 1 |

Qualitative variables are represented in percentage while quantitative variables are represented as mean ± standard error mean. P value refers to the comparation between naive and INSTIs groups. Statistically significant p values are in bold. AIDS (acquired immunodeficiency syndrome), HS (heterosexual), INSTIs (integrase strand transfer inhibitors-based treatment), MSM (men who have sex with men).


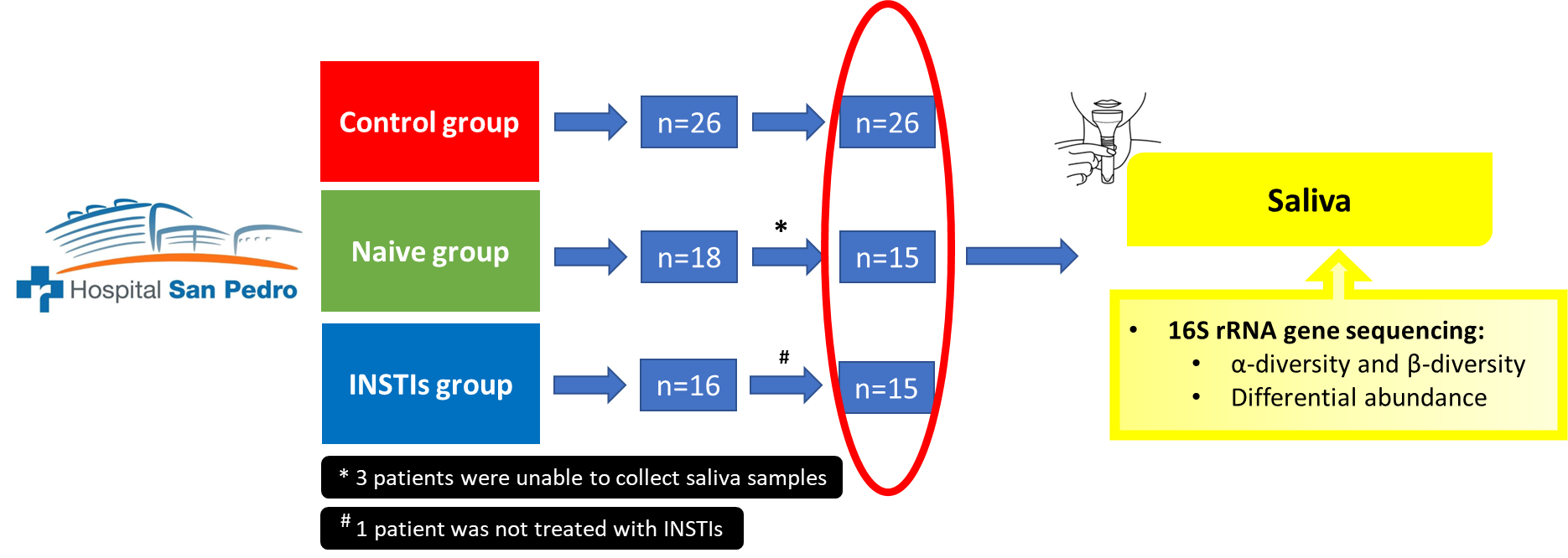
**Supplementary Figure 1.** Flowchart of patient recruitment. INSTIs (integrase strand transfer inhibitors-based treatment).


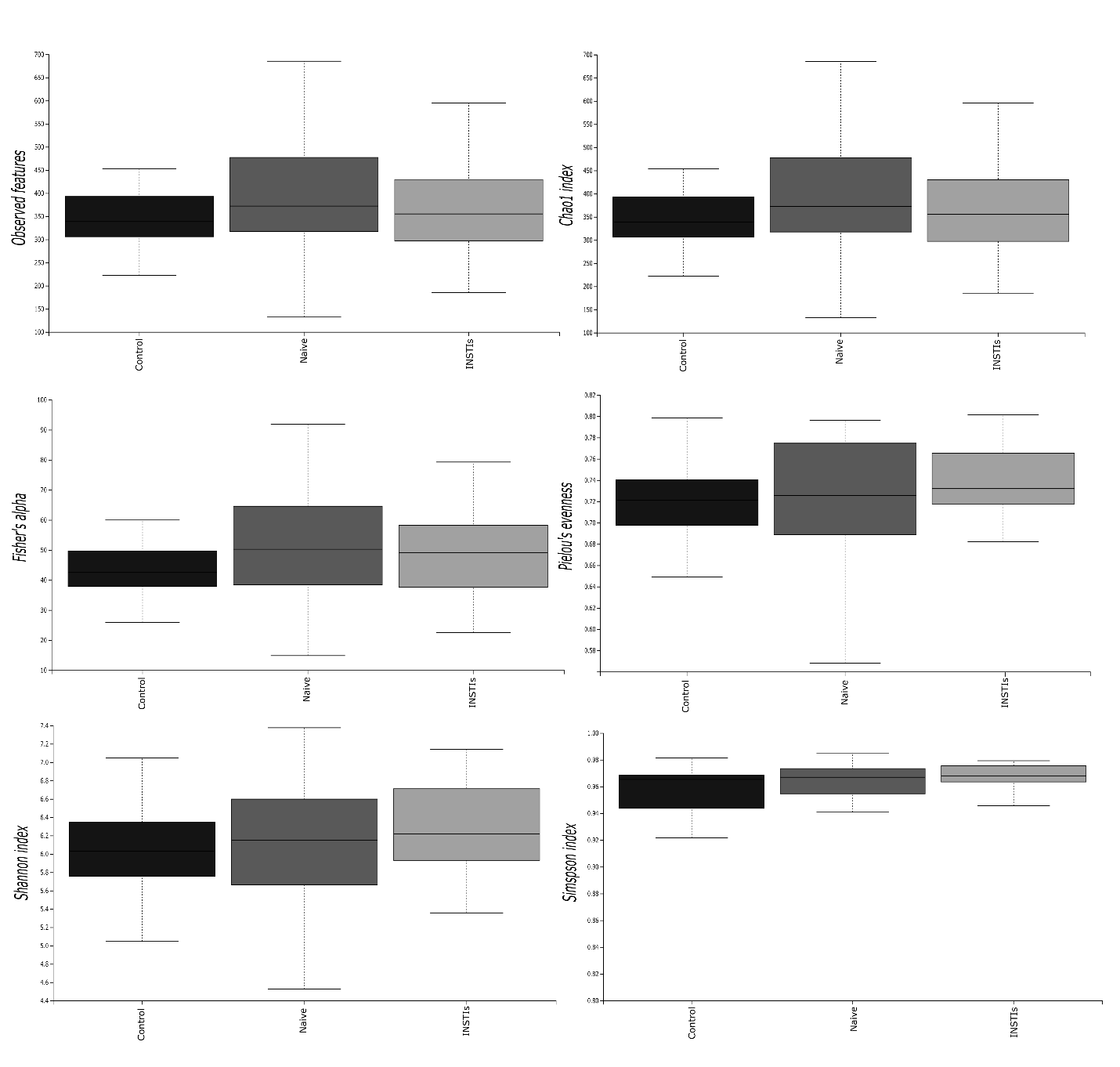
**Supplementary Figure 2.** Different indexes of α-diversity from bacteria in salivary samples of the studied population. INSTIs (integrase strand transfer inhibitors-based treatments).


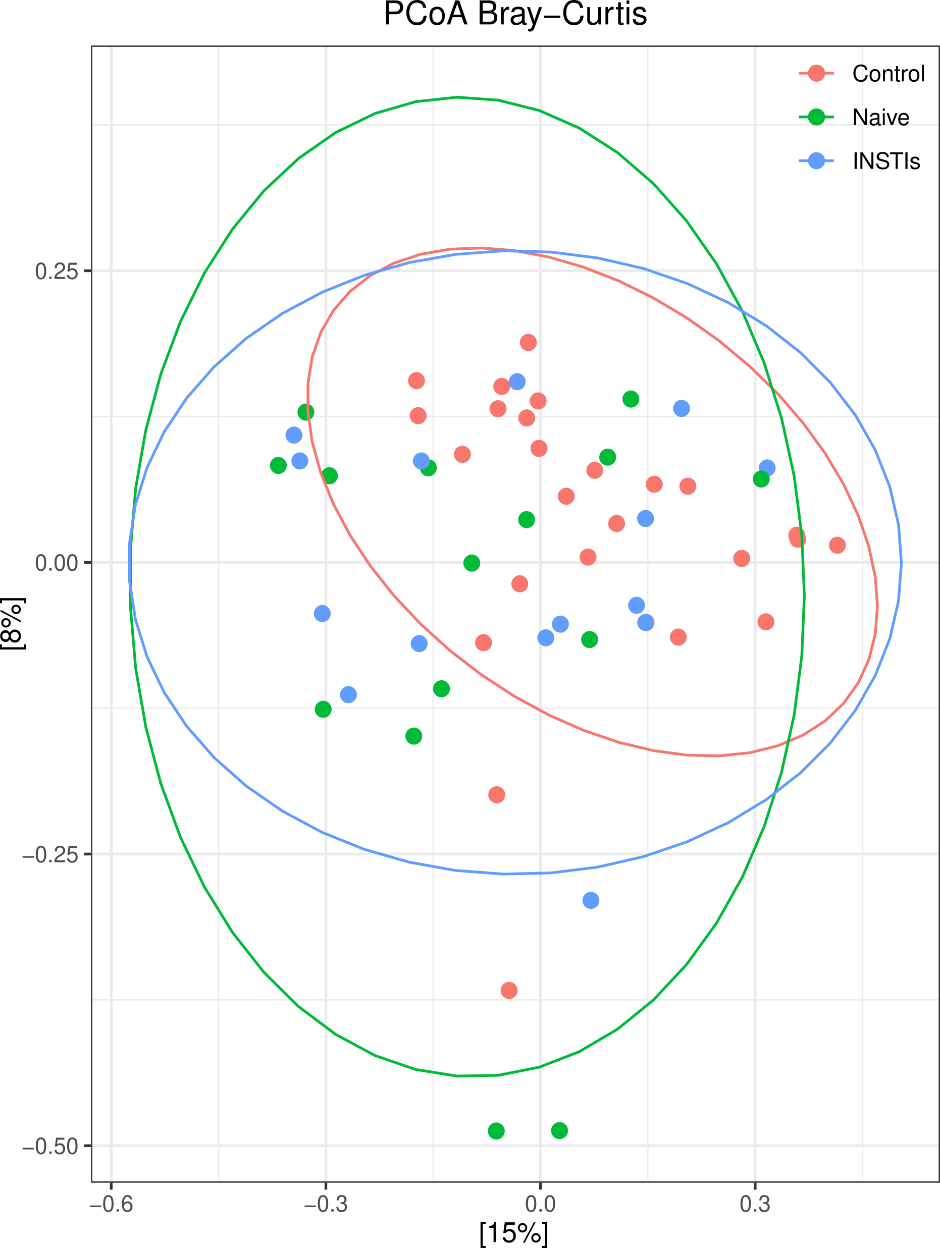
**Supplementary Figure 3.** PCoAs from bacteria in salivary samples from the studied population (accounting for 23% of the total variation [Component 1 = 15% and Component 2 = 8%]). Results are plotted according to the first two principal components. Each circle represents a sample: red circles represent the uninfected volunteers, green circles represent the naive group and blue circles represent the INSTIs-treated group. The clustering of samples is represented by their respective 95% confidence interval ellipse. INSTIs (integrase strand transfer inhibitors-based treatments).


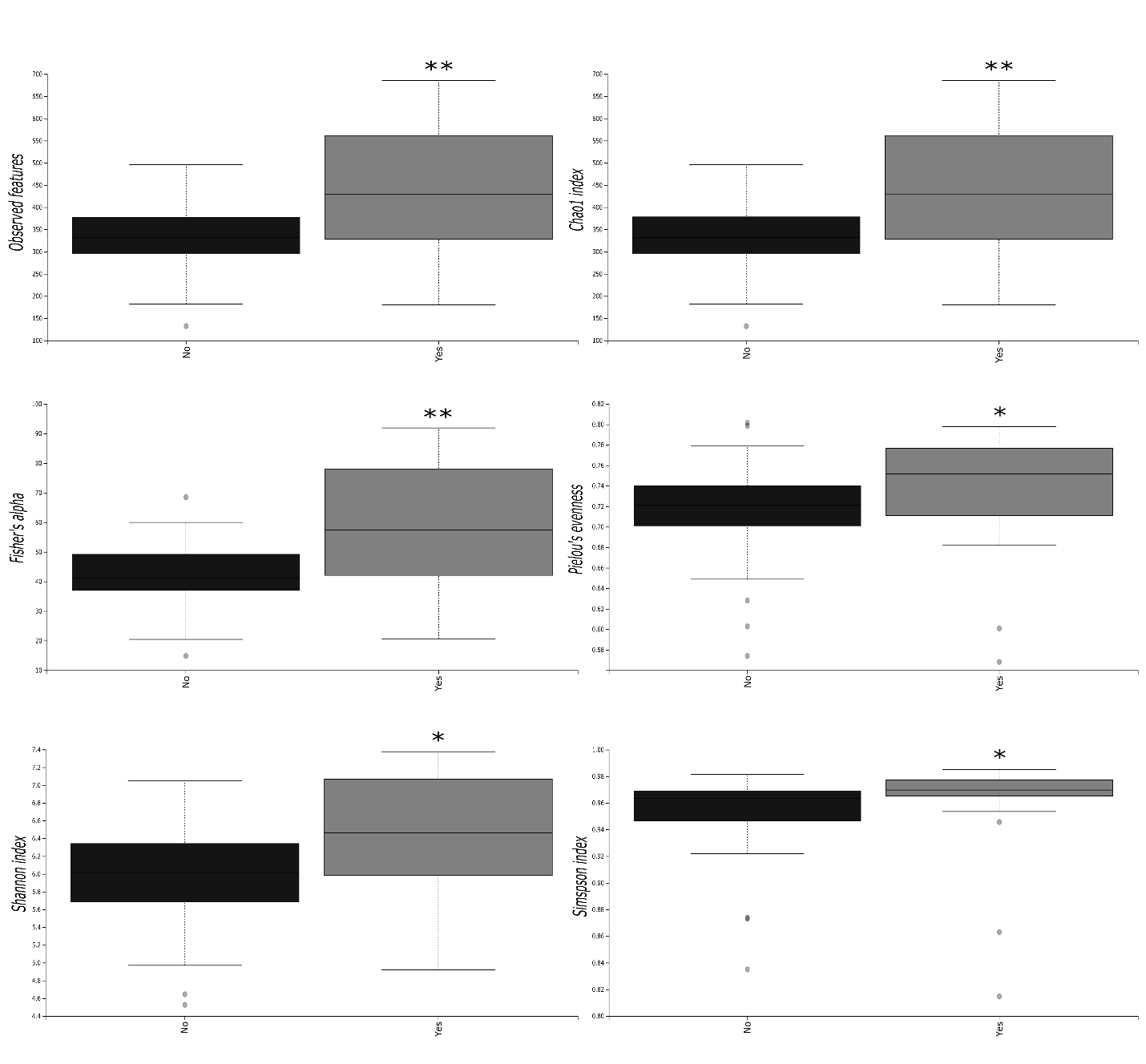
**Supplementary Figure 4.** Different indexes of α-diversity from bacteria in salivary samples of the studied population regarding smoking habit. *p<0.05 and **p<0.01 *vs.* non-smokers.


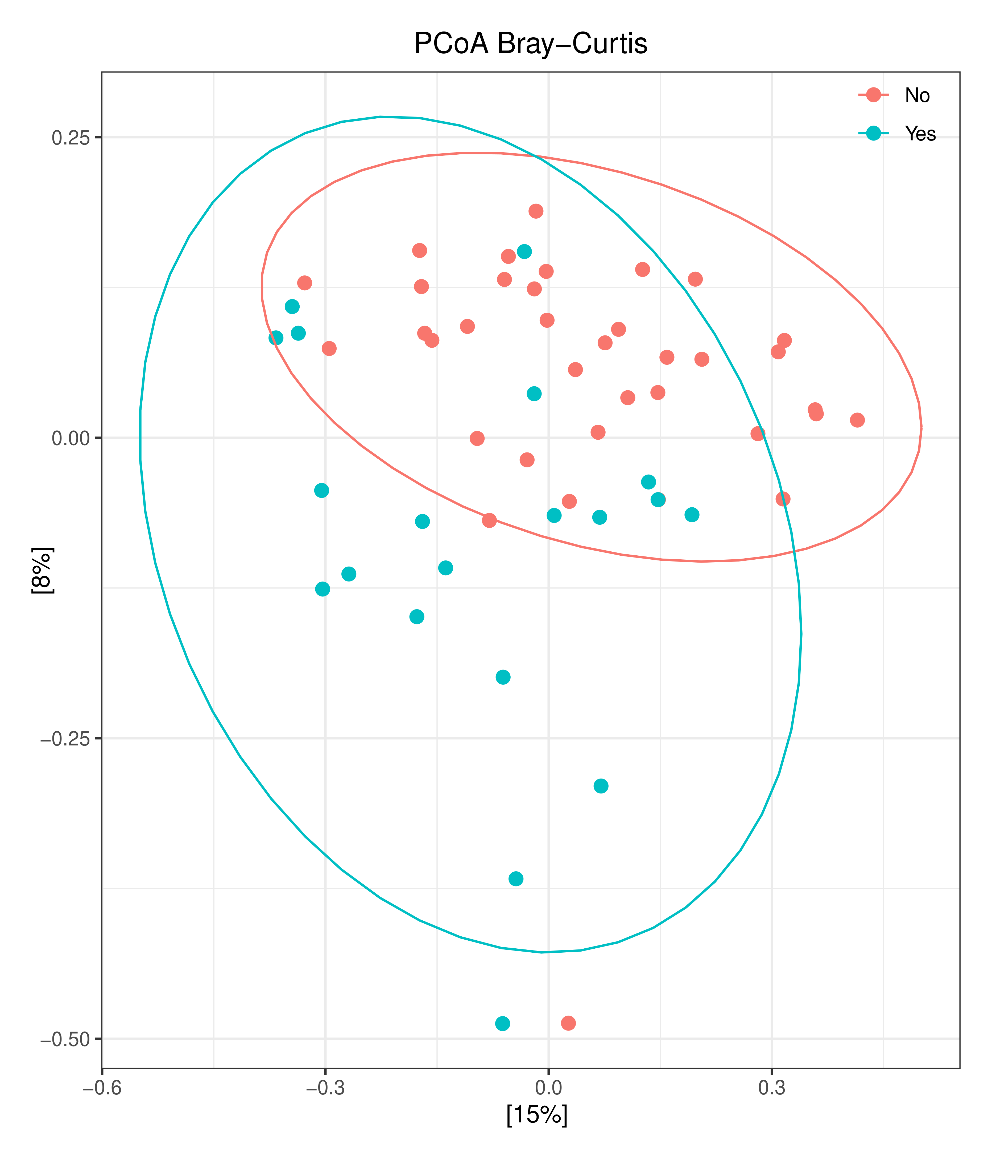
**Supplementary Figure 5.** PCoAs from bacteria in salivary samples from the studied population regarding smoking habit (accounting for 23% of the total variation [Component 1 = 15% and Component 2 = 8%]). Results are plotted according to the first two principal components. Each circle represents a sample: red circles represent the non-smoking individuals and blue circles represent the smoking individuals. The clustering of samples is represented by their respective 95% confidence interval ellipse. *p<0.001 smokers *vs.* non-smokers.


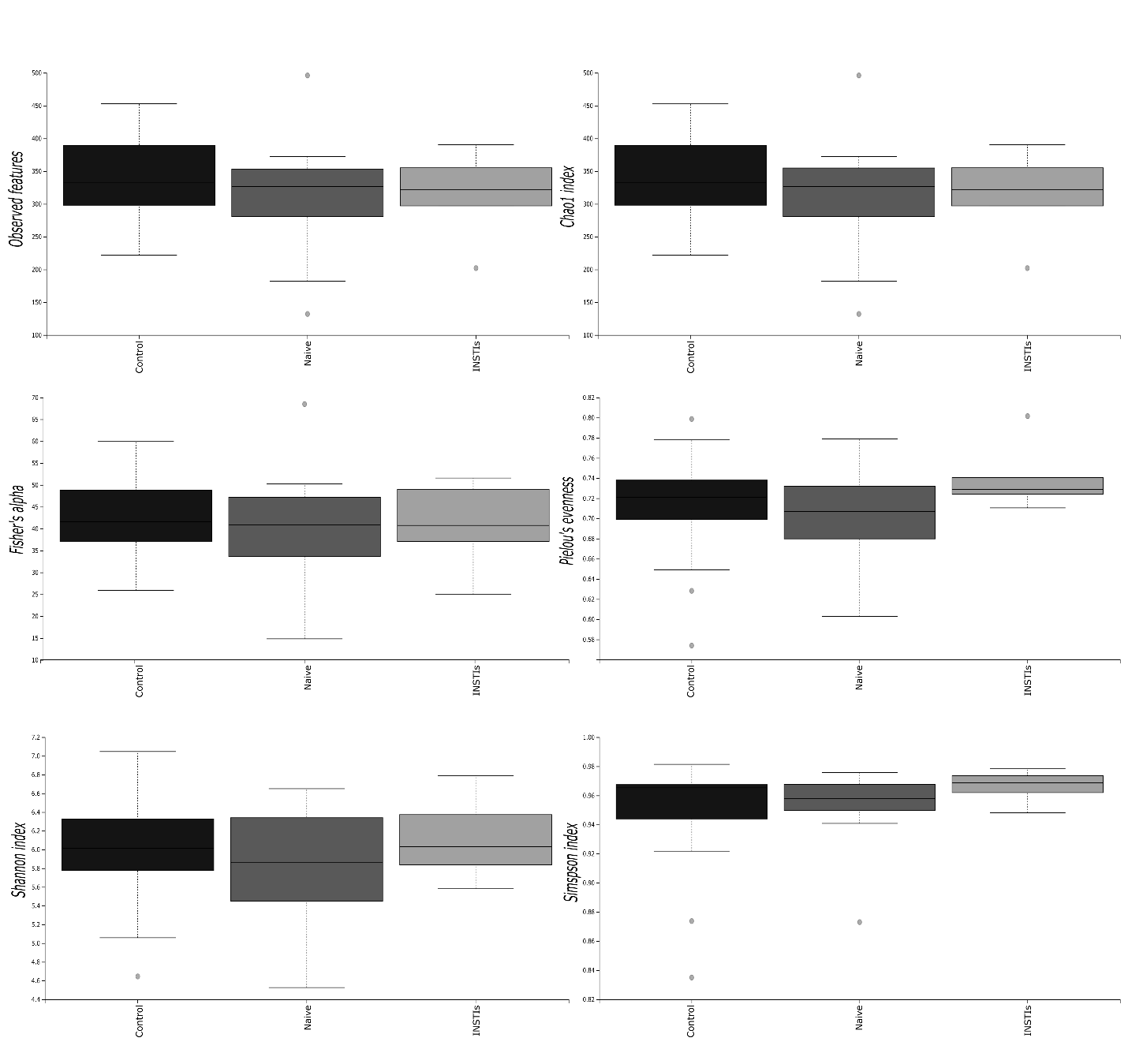
**Supplementary Figure 6.** Different indexes of α-diversity from bacteria in salivary samples of the non-smoking population. INSTIs (integrase strand transfer inhibitors-based treatment).


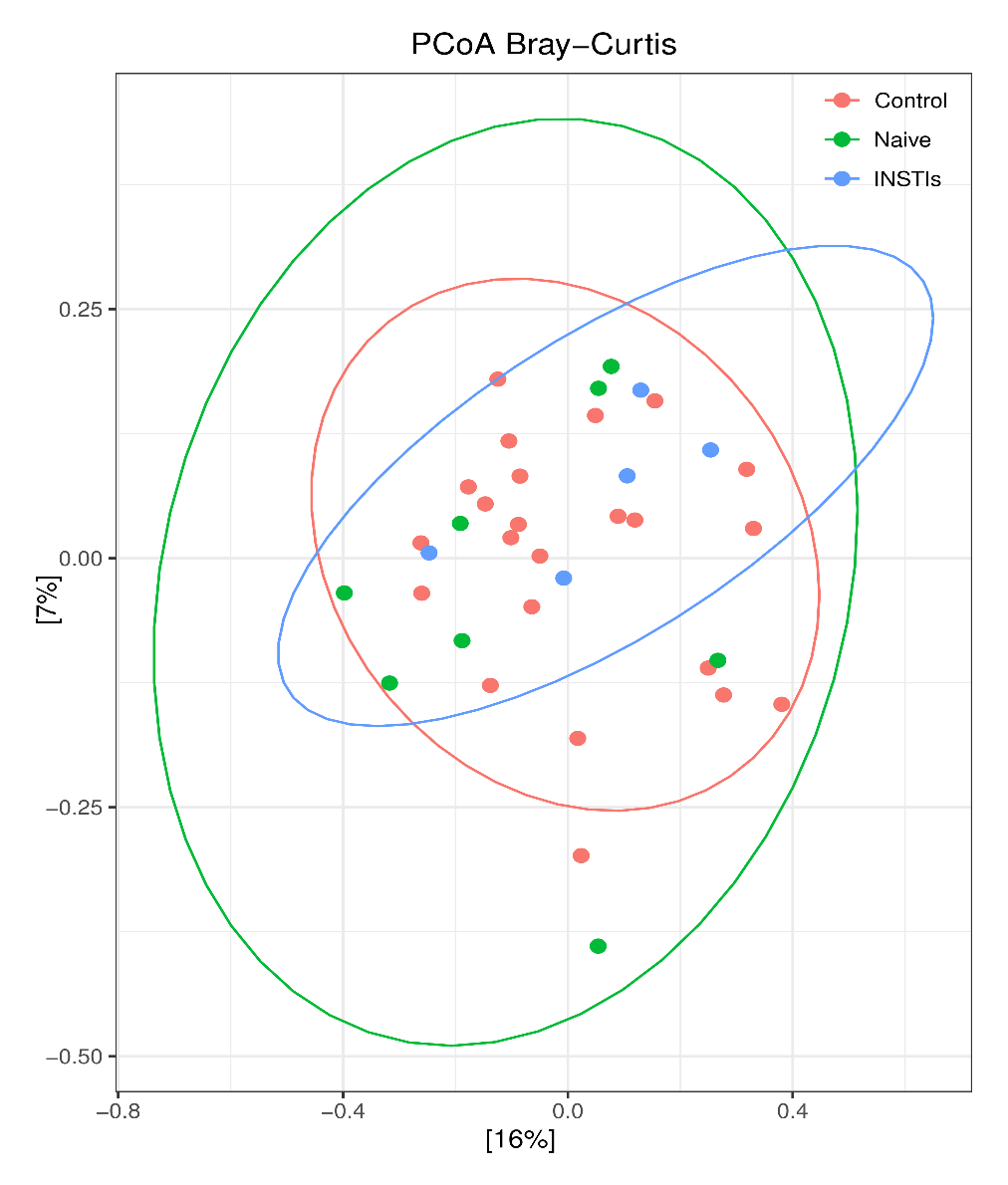
**Supplementary Figure 7.** PCoAs from bacteria in salivary samples from the non-smoking population (accounting for 23% of the total variation [Component 1 = 16% and Component 2 = 7%]). Results are plotted according to the first two principal components. Each circle represents a sample: red circles represent the uninfected volunteers, green circles represent the naive group and blue circles represent the INSTIs-treated group. The clustering of samples is represented by their respective 95% confidence interval ellipse. INSTIs (integrase strand transfer inhibitors-based treatments).


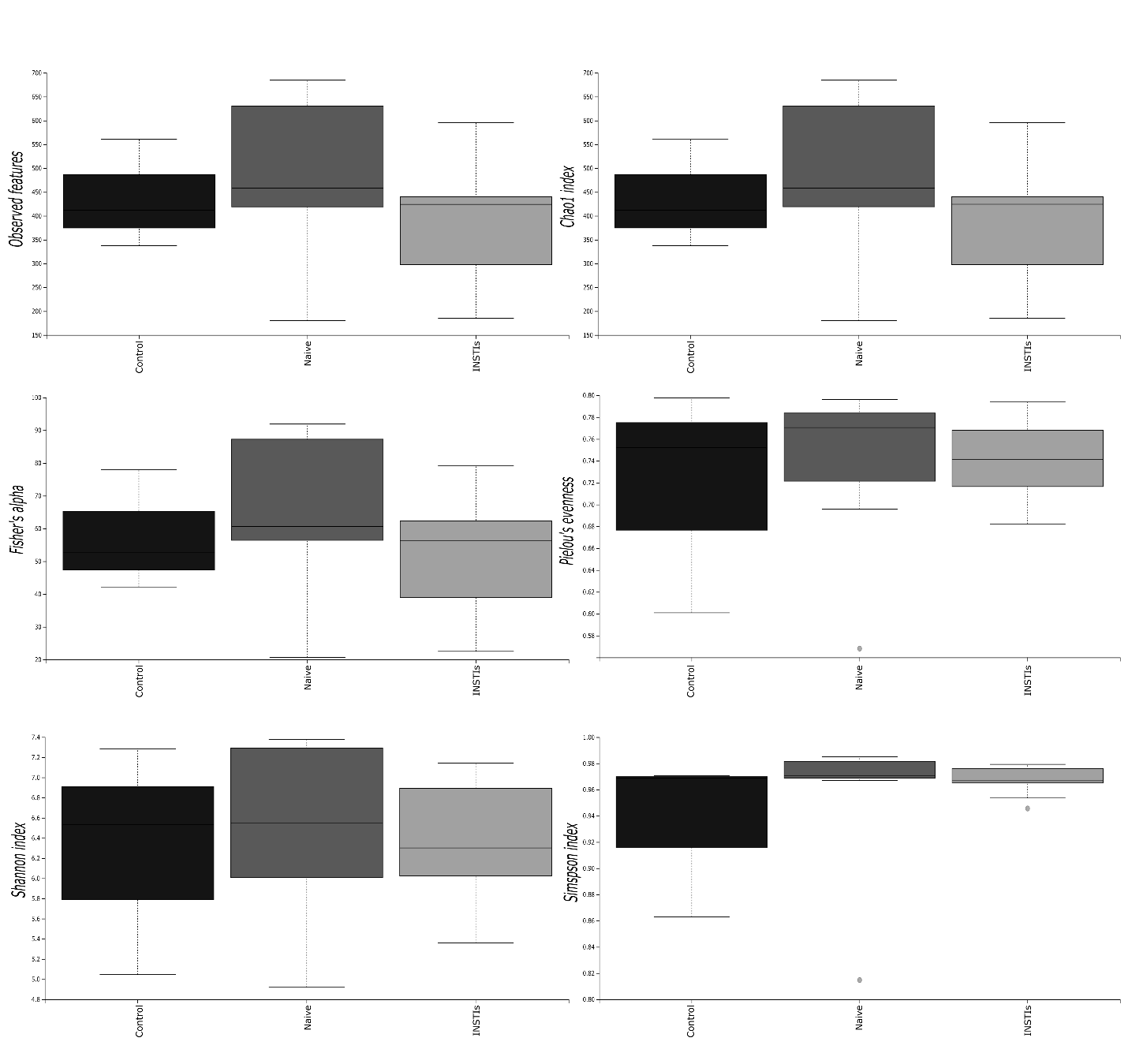
**Supplementary Figure 8.** Different indexes of α-diversity from bacteria in salivary samples of the smoking population. INSTIs (integrase strand transfer inhibitors-based treatment).


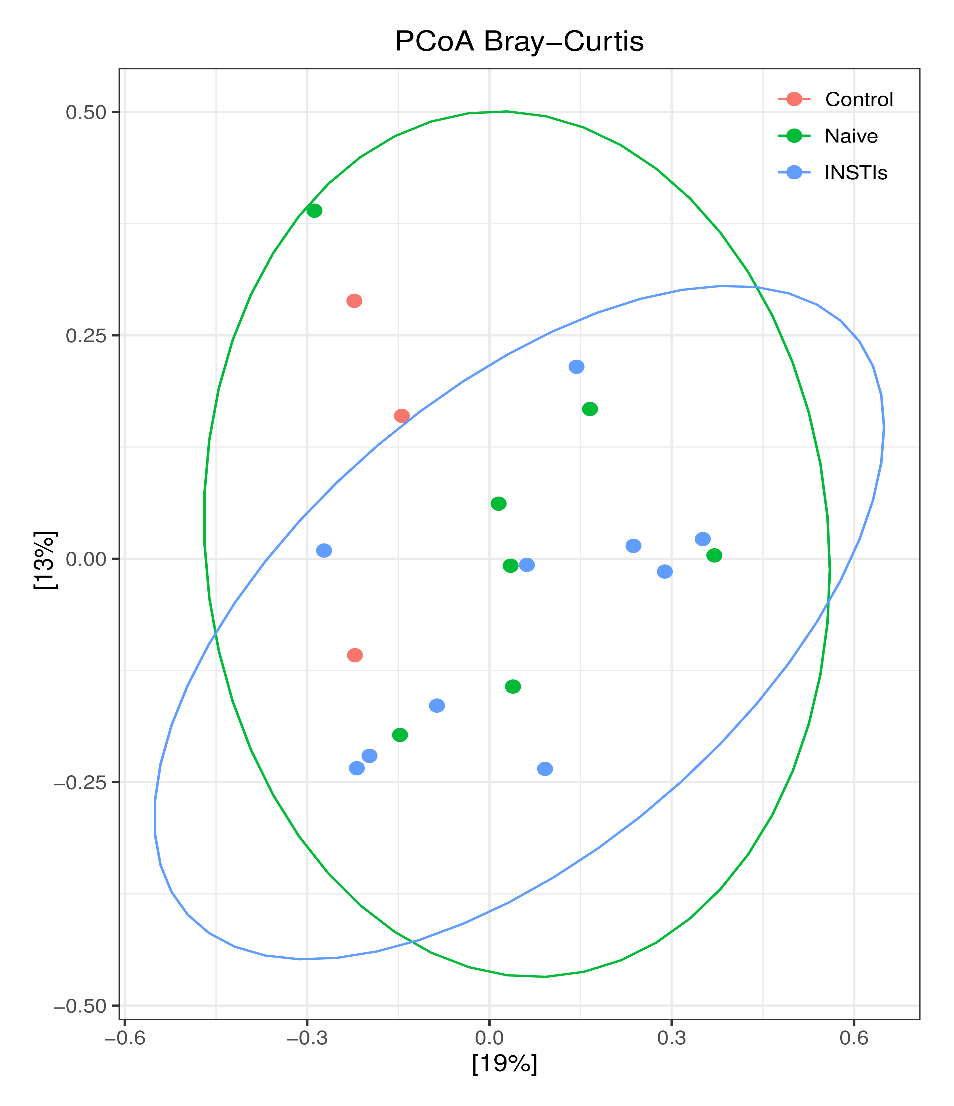
**Supplementary Figure 9.** PCoAs from bacteria in salivary samples from the smoking population (accounting for 32% of the total variation [Component 1 = 19% and Component 2 = 13%]). Results are plotted according to the first two principal components. Each circle represents a sample: red circles represent the uninfected volunteers, green circles represent the naive group and blue circles represent the INSTIs-treated group. The clustering of samples is represented by their respective 95% confidence interval ellipse. INSTIs (integrase strand transfer inhibitors-based treatments).
